# An increased fluid intake leads to feet swelling in 100-km ultra-marathoners - an observational field study

**DOI:** 10.1186/1550-2783-9-11

**Published:** 2012-04-03

**Authors:** Caroline Cejka, Beat Knechtle, Patrizia Knechtle, Christoph Alexander Rüst, Thomas Rosemann

**Affiliations:** 1 Institute of General Practice and for Health Services Research, University of Zurich, Zurich, Switzerland; 2 Gesundheitszentrum St. Gallen, St. Gallen, Switzerland

**Keywords:** Fluid intake, Peripheral oedemas, Sodium, Hydrations status, Ultra-marathon

## Abstract

**Background:**

An association between fluid intake and changes in volumes of the upper and lower limb has been described in 100-km ultra-marathoners. The purpose of the present study was (*i*) to investigate the association between fluid intake and a potential development of peripheral oedemas leading to an increase of the feet volume in 100-km ultra-marathoners and (*ii*) to evaluate a possible association between the changes in plasma sodium concentration ([Na^+^]) and changes in feet volume.

**Methods:**

In seventy-six 100-km ultra-marathoners, body mass, plasma [Na^+^], haematocrit and urine specific gravity were determined pre- and post-race. Fluid intake and the changes of volume of the feet were measured where the changes of volume of the feet were estimated using plethysmography.

**Results:**

Body mass decreased by 1.8 kg (2.4%) (*p *< 0.0001); plasma [Na^+^] increased by 1.2% (*p *< 0.0001). Haematocrit decreased (*p *= 0.0005). The volume of the feet remained unchanged (*p *> 0.05). Plasma volume and urine specific gravity increased (*p *< 0.0001). Fluid intake was positively related to the change in the volume of the feet (*r *= 0.54, *p *< 0.0001) and negatively to post-race plasma [Na^+^] (*r *= -0.28, *p *= 0.0142). Running speed was negatively related to both fluid intake (*r *= -0.33, *p *= 0.0036) and the change in feet volume (*r *= -0.23, *p *= 0.0236). The change in the volume of the feet was negatively related to the change in plasma [Na^+^] (*r *= -0.26, *p *= 0.0227). The change in body mass was negatively related to both post-race plasma [Na^+^] (*r *= -0.28, *p *= 0.0129) and running speed (*r *= -0.34, *p *= 0.0028).

**Conclusions:**

An increase in feet volume after a 100-km ultra-marathon was due to an increased fluid intake.

## Background

Participation in ultra-marathon running is of increasing popularity [1-5] where an ultra-marathon is a running race longer than the marathon distance of 42.195 km [5]. Within the ultra-marathons, there is a difference between single stage races [1,2,6,7] and multi-stage races [3,5], where the distance is split into daily stages. Running an ultra-marathon is associated with different problems such as a change in body mass [1,8-10], dehydration [10], a loss of skeletal muscle mass [3,7], an increase in total body water [3,4,6,11], overuse injuries of the lower limbs with especially knee injuries [5] and an impaired renal function due to exertional rhabdomyolysis [7], leading in extreme cases to a renal failure [12].

Among these ultra-running associated problems, an increase in total body water has been reported [3,4,6,11] and the development of peripheral oedemas has been described in this context in endurance athletes [4,13,14]. In single stage ultra-distance races, Stuempfle et al. [15] reported a fluid overload caused by excessive fluid consumption during cold weather in a 161-km race in Alaska leading to both an increase in plasma volume and a decrease in serum sodium concentration ([Na^+^]). A decreased serum [Na^+^] as well as an increase in total body water has also been reported for male 100-km ultra-marathoners [6] and it was presumed that the increase in total body water led to the development of oedemas [6]. In contrast to male 100-km ultra-marathoners, total body water and serum [Na^+^] remained unchanged in female 100-km ultra-marathoners while drinking ad libitum [1]. Apart from ultra-running, also after a Triple Iron triathlon, both total body water and plasma volume increased and clinically visible oedemas of the feet persisted until four days after the finish of the race [4].

An increase in total body water has also been reported for ten male multi-stage ultra-marathoners competing over 1,200 km with 17 consecutive stages [3]. Presumably, both the damage of skeletal muscle leading to rhabdomyolysis and an impaired renal function was the main factor for this accumulation of body water, since these ultra-runners suffered a decrease of skeletal muscle mass [3]. Exertional rhabdomyolysis due to exercise-induced myoglobinuria has been described before [7,12]. In another multi-stage ultra-endurance exercise of five consecutive days of hill walking, an increase in leg volume in five male subjects due to fluid and sodium retention has been described [13]. These authors reported an increase in aldosterone activity leading to an increase in serum [Na^+^], fluid retention and an increased shift of fluid from the intracellular to the extracellular fluid compartment. Similar findings on fluid homeostasis have been reported for five subjects during seven consecutive days of hill-walking, where facial and ankle oedemas also due to a retention in plasma [Na^+^] and a shift of water from the intracellular to the extracellular space with an expansion of the extracellular volume have been observed [14]. Therefore, a mechanism leading to an increase in total body water and a subsequent development of peripheral oedemas could be an increase of plasma volume due to [Na^+^] retention [11,13,14] as a consequence of an increased activity in plasma aldosterone [13,16] in response to an endurance exercise [16].

However, another potential mechanism leading to an increase in total body water might be fluid overload. In case of excessive fluid intake with fluid overload [17-19], we would expect an increase in total body mass [17,19,20] with a decrease in plasma [Na^+^] [17-21], an increase in plasma volume and a decrease in haematocrit due to haemodilution [15]. An inverse relationship between the percentage body mass loss during an endurance race and post-race serum [Na^+^] has been reported in several studies [17,20,22-26], where athletes losing the least amount of body mass or even gaining body mass during a race showed the lowest post-race serum [Na^+^], indicating that exercise-associated hyponatremia (EAH) is associated with minimal body mass loss or body mass gain [20,23]. This is consistent with the observation that fluid overload due to excessive fluid consumption is the main risk factor for EAH [19-21], which is defined as serum [Na^+^] < 135 mmol/l during exercise or up to 24 h after exercise [27]. Since ultra-marathoners are competing at a low intensity and have many aid stations during the race [1,9], they are at a higher risk for overdrinking [9,26] and subsequently developing EAH [19-21]. Besides fluid overload and plasma [Na^+^] retention due to an increased aldosterone activity, additional mechanisms could lead to a retention in total body water in ultra-endurance athletes such as protein catabolism and subsequent development of hyperproteinemic oedemas [28], an increased plasma volume due to an increased protein synthesis [29,30], an increased plasma volume due to an increased activity in vasopressin [31] or impaired renal function due to skeletal muscle damage [3,7,12].

Since there are several different mechanism described in the literature, which may lead to a retention of total body water and may lead to a potential development of peripheral oedemas, a recent field study investigated a potential association between both fluid and electrolyte intake and the formation of peripheral oedemas in 50 male 100-km ultra-marathoners [32]. The main finding was that total fluid intake was positively related to the changes in the volumes of both the upper and the lower limb, where athletes with an increased fluid intake developed an increase in the limb volumes. The authors found no association between fluid regulating hormones (i.e. copeptin and aldosterone) and renal parameters with the changes in limb volumes and concluded that fluid overload was the most likely mechanism leading to an increase in limb volumes. However, in that study, the volume of both the lower leg and the arm using plethysmography showed no changes whereas the thickness of adipose subcutaneous tissue at the hands and feet increased using the LIPOMETER^®^. The authors presumed that these disparate findings were due to a redistribution of the limb volume limited to hands and feet and not involving the whole limb [32].

Basing upon these recent findings, we might assume that an increased fluid intake may lead to an increase in feet volume. To our knowledge, there have been no studies to date investigating a potential association between changes in the feet volume and fluid intake in a 100-km ultra-marathon. The aims of the present study were, therefore, to investigate in 100-km ultra-marathoners (*i*) whether peripheral oedemas leading to an increase of the feet volumes would occur and (*ii*) in case of measurable increases, whether fluid overload would be associated with these increases. We hypothesized (*i*) that an ultra-marathon would lead to peripheral oedemas with an increase in the feet volume and (*ii*) that fluid overload would be associated with this increase. In case of fluid overload leading to an increase in feet volume, we hypothesized (*iii*) that there would be an association between the changes in plasma [Na^+^] and feet volume and that an increased fluid intake would lead to both an increase in feet volume and a decrease in plasma [Na^+^], thus leading to an increased prevalence of EAH. To test this hypothesis, we investigated a potential association between changes in feet volume using plethysmography with fluid intake in male 100-km ultra-marathoners.

## Methods

### Subjects

The organiser of the '100 km Lauf Biel' http://www.100km.ch in Biel, Switzerland, contacted all participants of the 2011 race three months before the start via a separate newsletter and informed them about the planned investigation. A total of 80 recreational male ultra-runners volunteered to participate in the study, 76 participants finished the race successfully within the time limit of 21 hours. The characteristics of anthropometry and training of the participants are presented in Table 1. The study was approved by the Institutional Review Board for the Use of Human Subjects of the Canton of St. Gallen, Switzerland, and all athletes gave their informed written consent.

**Table 1:** Characteristics of the subjects (*n *= 76).

*Characteristics*	*n*	*Result*
Age (years)	76	47.1 (8.6)
Body height (m)	76	1.80 (0.06)
Body mass (kg)	76	76.1 (9.8)
Body mass index (kg/m^2^)	76	23.4 (2.2)
Experience as ultra-runner (years)	76	12.3 (8.2)
Running training volume (h/week)	76	7.8 (8.9)
Running training volume (km/week)	76	66.2 (26.6)
Running training speed (km/h)	76	10.6 (1.7)
Marathons finished (number)	74	45 (98)
100-km ultra-marathons finished (number)	52	6 (6)
Personal best marathon time (min)	74	211 (33)
Personal best 100-km ultra-marathon time (min)	52	589 (297)

Results are presented as mean and SD

### The race

The '100 km Lauf Biel' took place on June 17, 2011. The athletes started the 100-km road course ultra-marathon at 10:00 p.m. During these 100 km with a total change in altitude of ~645 metres, the organiser provided a total of 17 aid stations offering an abundant variety of food and beverages such as hypotonic sports drinks, tea, soup, caffeinated drinks, water, bananas, oranges, energy bars and bread. The athletes were allowed to be supported by a cyclist in order to have additional food and clothing, if necessary. The temperature at the start was 21°C, dropping to 12°C during the night and rising to 13°C the morning of the next day. At the start, there was no rain. During the night, there were some showers.

### Measurements and calculations

On June 17, 2011, between 05:00 p.m. and 10.00 p.m., the pre-race measurements were performed. Body mass was measured using a commercial scale (Beurer BF 15, Beurer GmbH, Ulm, Germany) to the nearest 0.1 kg after voiding of the urinary bladder. Capillary blood samples were drawn from the fingertip. Plasma sodium [Na^+^] and haematocrit were analysed using the i-STAT^® ^1 System (Abbott Laboratories, Abbott Park, IL, USA). Standardisation of posture prior to blood collection was respected since postural changes can influence blood volume and therefore haematocrit [33]. The percentage change in plasma volume was calculated from pre- and post-race values of haematocrit following the equation of van Beaumont [34]. Urine specific gravity was analysed using Clinitek Atlas^® ^Automated Urine Chemistry Analyzer (Siemens Healthcare Diagnostics, Deerfield, IL, USA).

The volume and the changes of volume of the right foot were measured using the principle of plethysmography. We used a Plexiglas^® ^vessel with the internal dimensions of 386 mm length and 234 mm width. These dimensions were chosen so that any foot size of a male runner would fit in the vessel. Outside the vessel, a scale in mm was fixed on the front window measuring changes in the level of water from the bottom to the top. The vessel was filled to the level of 100 mm with plain water. At 100 mm, the complete food was immersed in the water and the upper limit of the water was at the middle of *malleolus medialis*. After immersion of the foot, the new water level was recorded to the nearest 1 mm. With the dimension of length (386 mm), width (234 mm) and height (displaced water level in mm), the volume of the foot was estimated. The corresponding calculated volume in mL using the length, width and height in mm of the displaced water was defined as the volume of the right foot. The reproducibility of the applied method of water displacement using the changes in height in mm was evaluated in a separate series of 20 consecutive measurements in one individual. The coefficient of variance (CV) was 1.9%; the mean height of displaced water was 12.0 mm, the 95*% *confidence interval was 11.8-12.1 mm, and the standard error was 0.05. The CV of the pre-race measurements (*n *= 76) was 20.2%, the CV of the post-race measurements was 20.5%.

Immediately after arrival at the finish line, the identical measurements were repeated. Between the pre-race and post-race measurements, the athletes recorded their intake of food and drinks using a prepared paper and pencil. At each of the 17 aid station they noted both the kind and the amount of ingested food and fluids. At these aid stations, liquids and food were prepared in a standardized manner, i.e. beverages and food were provided in standardized size portions. The drinking cups were filled to 0.2 L; the energy bars and the fruits were halved. The athletes also recorded additional food and fluid intake provided by their support crew, as well as their intake of salt tablets and other supplements. The compositions of fluids and solid food were estimated using a food table [35].

### Statistical analysis

Data are presented as mean and standard deviation (SD). Pre- and post-race results were compared using paired *t*-test. Pearson correlation analysis was used to check for associations between the measured and calculated parameters. Statistical significance was accepted with *p *< 0.05 (two-sided hypothesis).

## Results

**Figure 1:**
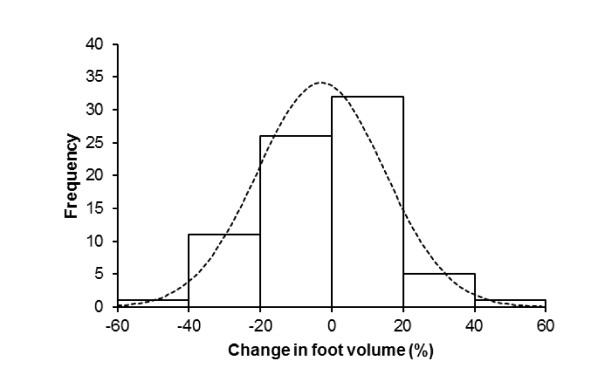
**Range of changes in foot volume**.

Seventy-six of the 80 subjects completed the 100-km ultra-marathon within 731 (130) min, running at an average speed of 8.4 (1.4) km/h. Their training and previous experience is presented in Table 1. Four subjects failed to finish the 100-km race due to overuse injuries of the lower limbs and were withdrawn from the study. Table 2 shows the pre- and post-race measurements and their changes. Body mass decreased significantly by 1.8 (1.4) kg from 76.1 (9.8) kg pre-race to 74.3 (9.9) kg post-race (*p *< 0.0001), representing a 2.4% decrease in body mass. The volume of the foot remained unchanged (*p *> 0.05). In detail: in 20 runners, the foot volume increased, in 18 runners the volume showed no change and in 38 runners foot the volume decreased (Figure 1).

**Table 2:** Results of the physical, haematological and urinary parameters before and after the race.

	Pre-race*	Post-race*	Absolute change*	Percent change*	*p*-value**
Body mass (kg)	76.1 (9.8)	74.3 (9.9)	-1.8 (1.4)	-2.4 (1.8)	< 0.0001
Volume of the right foot (mL)	1,118 (225)	1,073 (227)	-45 (201)	-2.7 (18.2)	> 0.05
Haematocrit (%)	44.8 (3.3)	43.6 (2.9)	-1.2 (2.7)	-2.3 (5.8)	0.0005
Plasma [Na^+^] (mmol/l)	137.0 (2.7)	138.6 (2.6)	+1.6 (3.1)	+1.2 (2.3)	< 0.0001
Urine specific gravity (g/ml)	1.015 (0.008)	1.024 (0.008)	+0.009 (0.008)	+0.87 (0.79)	< 0.0001

* *n *= 76, mean and (SD), ** by paired *t*-test

**Figure 2:**
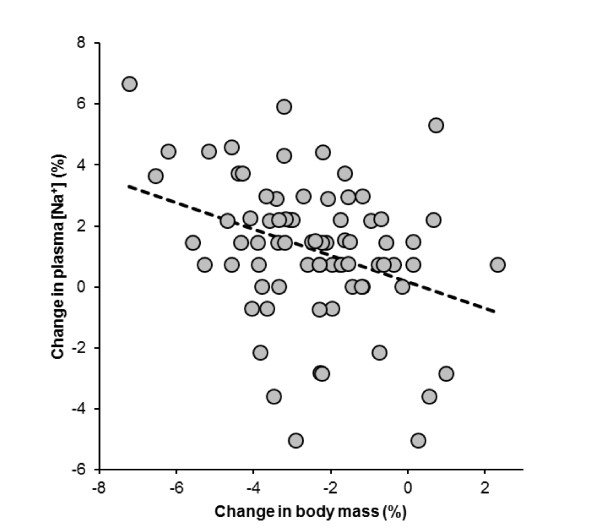
**The change in body mass was significantly and negatively related to the change in plasma [Na^+^] (*r *= -0.35, *p *= 0.0023)**.

**Figure 3:**
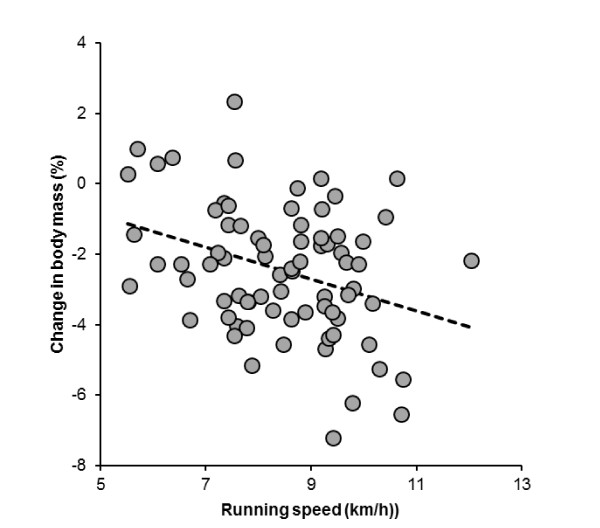
**The change in body mass was significantly and negatively related to running speed (*r *= -0.34, *p *= 0.0028)**.

Haematocrit decreased (*p *= 0.0005), plasma volume increased by 5.3% (11.9) and urine specific gravity increased (*p *< 0.0001). Plasma [Na^+^] increased significantly (*p *< 0.0001) by 1.2% from 137.0 (2.7) mmol/l to 138.6 (2.67) mmol/l, with a mean difference of 1.6 (3.1) mmol/l. Pre-race, 10 subjects showed plasma [Na^+^] < 135 mmol/L with values between 131 mmol/L and 134 mmol/L. Post-race, four subjects (5.3%) developed asymptomatic EAH with post-race plasma [Na^+^] between 132 mmol/L and 134 mmol/L. The lowest post-race plasma [Na^+^] was 132 mmol/L in these subjects. Pre-race plasma [Na^+^] in these four subjects was 139 mmol/L. Table 3 summarizes their pre- and post-race values, fluid intake and foot volume changes. Two subjects had both pre-and post-race plasma [Na^+^] < 135 mmol/L, with a pre-race plasma [Na^+^] of 133 mmol/l in one subject, and 131 mmol/L in the other subject, respectively. The change in body mass was significantly and negatively related to the change in plasma [Na^+^] (Figure 2) and running speed (Figure 3), respectively.

**Table 3:** Data for each individual who was hyponatremic post-race

Subject	Pre-race plasma [Na^+^] (mmol/L)	Post-race plasma [Na^+^] (mmol/L)	Change in plasma [Na^+^] (mmol/L)	Fluid intake (L)	Change in foot volume (%)
1	139	132	- 7	3.0	- 30
2	139	132	- 7	20.0	+ 12.5
3	139	134	- 5	4.8	- 20
4	139	134	- 5	14.8	+ 8.3

**Figure 4:**
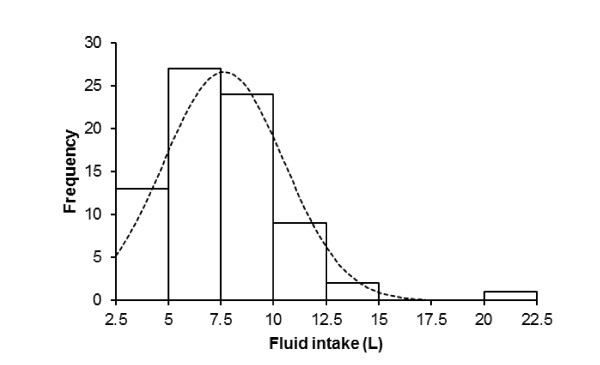
**Range of fluid intake**.

**Figure 5:**
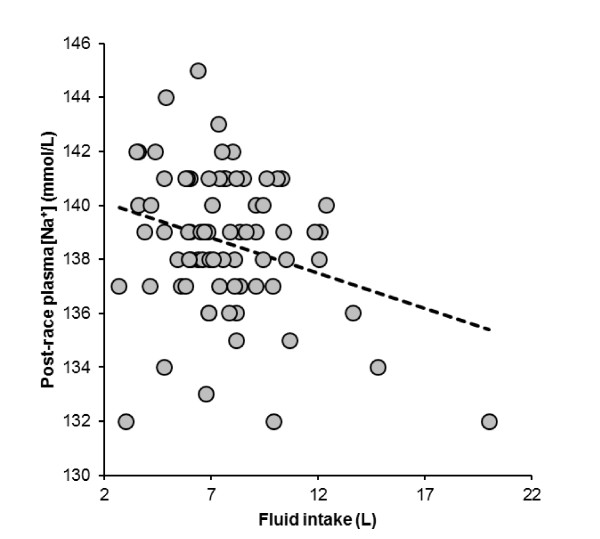
**Fluid intake was significantly and negatively related to post-race plasma [Na^+^] (*r *= -0.28, *p *= 0.0142)**.

**Figure 6:**
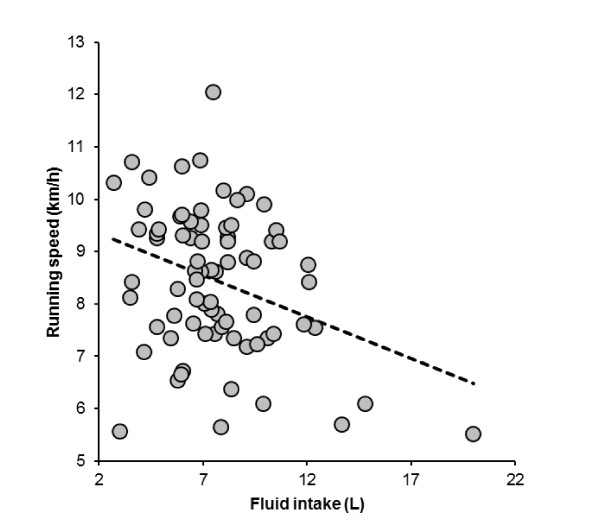
**Fluid intake was significantly and negatively related to running speed (*r *= -0.33, *p *= 0.0036)**.

The subjects consumed a total of 7.64 (2.85) L of fluids during the run, equal to 0.63 (0.20) L/h or 0.10 (0.03) L/kg body mass, respectively. Fluid intake varied between 2.7 L and 20 L (Figure 4). Fluid intake was significantly and negatively related to both post-race plasma [Na^+^] (Figure 5) and running speed (Figure 6), respectively, with faster athletes drinking less fluid while running. The change in plasma volume was associated with total fluid intake (*r *= 0.24, *p *= 0.04), but showed no association with the change in plasma [Na^+^].

**Figure 7:**
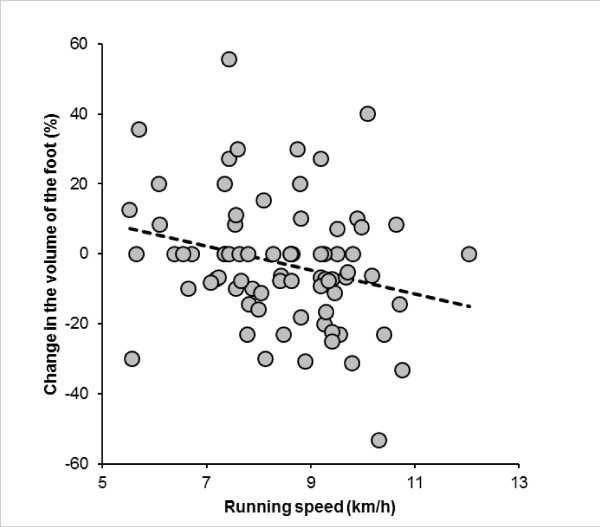
**The change in the volume of the right foot was significantly and negatively related to running speed (*r *= -0.23, *p *= 0.0236)**.

**Figure 8:**
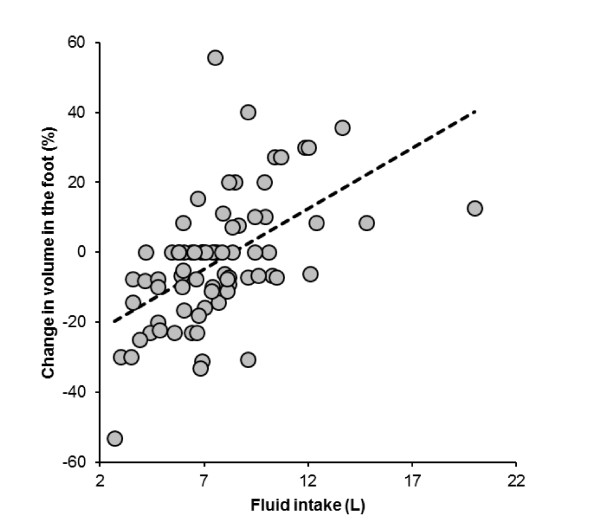
**Fluid intake was significantly and positively related to the change in the volume of the right foot (*r *= 0.54, *p *< 0.0001)**.

**Figure 9:**
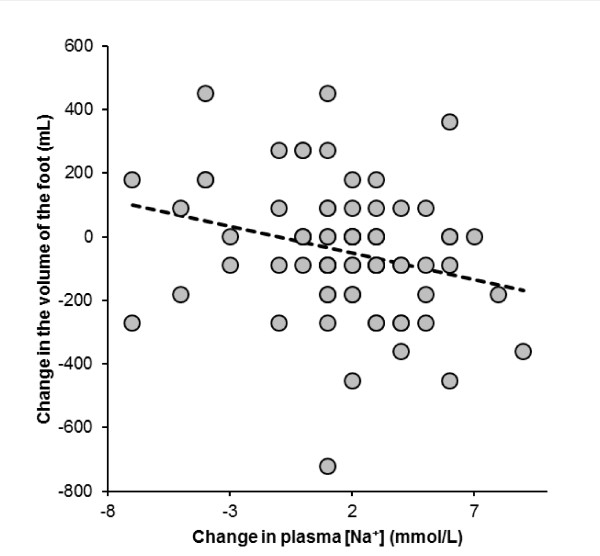
**The change in the volume of the right foot was significantly and negatively related to the change in plasma [Na^+^] (*r *= -0.26, *p *= 0.0227)**.

Running speed was significantly and negatively related to the change in the foot volume, whereas the volume of the foot tended to decrease in faster runners (Figure 7). Although the volumes of the foot showed no changes during the race, total fluid intake during the race was significantly and positively related to the change in the volume of the foot (Figure 8). The change in the volume of the foot was significantly and negatively related to the change in plasma [Na^+^] (Figure 9).

## Discussion

The first aim of the present study was to investigate whether peripheral oedemas leading to an increase of the feet volumes would occur in 100-km ultra-marathoners. Based on the existing literature, we hypothesized that an ultra-marathon can lead to peripheral oedemas with an increase in the feet volume and that fluid overload would be associated with these increases. In case of fluid overload leading to an increase in the feet volume we hypothesized furthermore finding an association between changes in plasma [Na^+^] and the feet volume and a higher prevalence of EAH. In accordance with our hypothesis, fluid intake was related to the change in feet volume. Furthermore, we found an association between the change in plasma [Na^+^] and the change in the feet volume. In addition, four subjects (5.3%) developed asymptomatic EAH with plasma [Na^+^] between 132 and 134 mmol/L.

The most important finding in this study was that fluid intake was significantly and positively related to the change in the foot volume, where an increased fluid intake was leading to an increased volume of the foot. Both the change in the foot volume and fluid intake were significantly and negatively related to running speed. Faster runners were drinking less during the race, and their foot volume tended to decrease. In accordance with our findings, Bracher et al. [32] demonstrated that fluid intake was positively related with the changes in the limb volumes. Since these authors found no association between fluid-regulating hormones and renal parameters with the changes in limb volumes, they concluded that fluid overload was the most likely mechanism leading to an increase in limb volumes. As fluid intake was associated with the change in foot volume in the present study, we assume that no significant changes in total body water occurred in the present participants responsible for a possible development of peripheral oedemas.

In case of excessive fluid intake with fluid overload, we would expect an increase in total body mass [17,19,20], a decrease in plasma [Na^+^] [17-21], an increase in plasma volume and a decrease in haematocrit due to haemodilution [15]. In the present subjects, haematocrit decreased significantly and plasma volume increased by 5.3% indicating that fluid overload occurred. However, body mass decreased, and both urine specific gravity and plasma [Na^+^] increased. In case of dehydration, as has been demonstrated in 12- and 24-hour ultra-marathoners [10], both a decrease in body mass and an increase in urine specific gravity have been reported [36,37]. Furthermore, an increase in plasma [Na^+^] is expected when the water loss, including water loss by sweat, inducing dehydration is hypotonic compared with plasma [37]. The present subjects lost 2.4% of their body mass during the race, which was equal to mild dehydration and their post-race urine specific gravity was 1.024 g/ml indicating even a significant dehydration according to Kavouras [36]. However, the change in urine specific gravity was very small and both pre-and post-race measurements were within the normal range limits [38]. Rather in contrast to the study of Kavouras [36], both Speedy et al. [23] and Rogers et al. [39] suggested that a part of the body mass loss during an ultra-endurance race could be the result of the metabolic breakdown of fuel, which includes a loss of fat, glycogen and water stored with glycogen. Speedy et al. [23] concluded that athletes lost 2.5 kg of body mass during an ultra-distance triathlon most likely from sources other than fluid loss. Thus, Speedy et al. [23,40] suggested that athletes who maintain their pre-race body mass or who sustain a minimal body mass loss may be either euhydrated or moderately overhydrated. Since the present athletes lost 1.8 kg of their body mass during an ultra-marathon, this could be due to other sources than fluid loss following Speedy et al. [23] and not indicate dehydration.

Recently, Hew-Butler et al. [41] reported that body mass was not an accurate surrogate of fluid balance homeostasis during prolonged endurance exercise. In their study of 181 male Ironman triathletes, despite significant body mass loss of 5% during the race, plasma volume and serum [Na^+^] were maintained. Thus, Hew-Butler et al. [41] concluded that the body protects osmolality in plasma and circulating blood volume during prolonged endurance exercise and this results in a net body mass loss. Similar findings were recently reported by Tam et al. [8] and these authors concluded that a reduction in body mass can occur without an equivalent reduction in total body water during prolonged exercise and that the body primarily defends plasma [Na^+^] and not body mass during exercise. In addition, Nolte et al. [42] recently suggested that a 1 kg loss in body mass in a 25-km route march in dry heat was associated with only a 200 g loss in total body water and concluded that changes in body mass did not accurately predict changes in total body water. In the present subjects, body mass decreased by 2.4%, plasma volume increased by 5.3% and post-race plasma [Na^+^] increased from 137.0 (2.7) mmol/l to 138.6 (2.67) mmol/l. Although the 1.6 (3.1) mmol/l increase in plasma [Na^+^] from pre-race to post-race was statistically significant, plasma [Na^+^] was still maintained within the normal range limits (135-145 mmol/L) [38]. An increase in plasma volume, despite a body mass loss has been documented in athletes competing in prolonged endurance events [13-15,23,41]. Hew-Butler et al. [41] suggested that there may be a 'fluid reserve' within the interstitial fluid of the extracellular fluid compartment in ultra-endurance athletes that could serve as a 'plasma volume reserve'. Fellman et al. [11] reported that prolonged and repeated exercise induced a chronic hyperhydration and that sodium retention was the major factor in the increase of plasma volume. Furthermore Milledge et al. [13] mentioned an increased activity of plasma aldosterone concentration responsible for the sodium retention. Since plasma [Na^+^] increased in the present subjects, this could be a possible reason for the increase in plasma volume. However, the change in plasma volume showed no correlation with the change in plasma [Na^+^] in the present subjects, but was associated with fluid intake. Presumably, the increase in plasma volume was due to fluid ingestion and there may be a potential internal water source, for example water previously stored with glycogen, that can be released during exercise and maintain blood biochemical parameters despite an absolute body weight loss [8,43]. Thus, the present results lead us to the conclusion that body fluid homeostasis was maintained in the present ultra-marathoners, despite a body mass loss of 2.4%. Accordingly, these actual data support the findings that the body primarily defends plasma [Na^+^] and circulating blood volume and not body mass during prolonged endurance exercises and that a change in body mass during exercise may not reflect exact changes in the hydration status [8,41].

A further finding was that four runners (5.3%) developed asymptomatic EAH with post-race plasma [Na^+^] between 132 and 134 mmol/L. Pre-race plasma [Na^+^] in these four subjects was 139 mmol/L. Two athletes showed plasma [Na^+^] < 135 mmol/l both pre-and post-race. By definition, no EAH occurred in this two subjects, since they both had a pre-race plasma [Na^+^] < 135 mmol/L. Overall, 10 subjects showed plasma [Na^+^] < 135 mmol/L with values between 131 mmol/L and 134 mmol/L pre-race. No symptomatic EAH occurred. The prevalence of 5.3% subjects with asymptomatic EAH in these 76 ultra-marathoners is rather low compared to other studies reporting prevalence of EAH in marathons and ultra-marathons between 0% and 51.2% [9,15,26,32,44]. Furthermore, we found a significant and negative correlation between post-race plasma [Na^+^] and the change in body mass; athletes who lost the least weight or even gained weight, had the lowest plasma [Na^+^] post- race. Our finding corresponds to results in several former studies [17,20,22-26], reporting a negative correlation between the change in body mass and post-race serum [Na^+^].

The present subjects showed a variation of total fluid intake between 2.7 and 20 L during the run with a mean fluid intake of 7.64 L, equal to 0.63 L/h. Fluid intake was significantly and negatively related to post-race plasma [Na^+^]. This result supports the findings of the existing data that EAH is associated with fluid overload [15,17-21,23]. To prevent excessive drinking during endurance exercise, the 'Position Statement of International Marathon Medical Directors Association' promotes that marathoners should drink according to their thirst, but no more than 0.4 to 0.8 L/h [45]. The present ultra-marathoners consumed on average 0.63 L/h, which corresponds to these recommendations. Paradoxically, one of the subjects who developed EAH post-race was also the subject who consumed fluid at one of the lowest rate with 0.28 L/h. This subject lost 2 kg (2.9%) body mass during the race, had a pre-race plasma [Na^+^] of 136 mmol/L and the foot volume decreased by 53%. He was also among the fastest runners finishing within 582 min (9 h 42 min). This result is not in line with our and other findings that a high fluid intake is correlated with lower post-race plasma [Na^+^] [17,19-21]. Possible explanations for this subject developing EAH could be other factors than excessive fluid consumption such as non-osmotic stimulation of arginine vasopressin (AVP) [31] or inability to mobilize osmotically inactive sodium from internal stores or inappropriate osmotic inactivation of circulating Na^+ ^[20]. Other possible reasons could be a loss of sodium. A loss of sodium could occur via urine if AVP had been present, or by sweat, or by some combination of these.

Finally, we found that the change in the foot volume was significantly and negatively related to the change in plasma [Na^+^]. As fluid intake was associated with the change in the foot volume, an increased fluid intake generally led to both a decrease in plasma [Na^+^] and an increase in the foot volume. Obviously, slower runners were drinking more and their post-race plasma [Na^+^] tended to decrease, since both fluid intake and the change in the feet volume was significantly and negatively related to running speed. In addition, slower runners showed an increase in the foot volume. Presumably, slower runners were sweating less and drinking at a higher rate than were the faster runners. As slower runners are more likely to overconsume fluids [26] and excessive fluid consumption is the main risk factor for EAH [19-21], we infer that fluid overload occurred in the slower runners. Thus, fluid overload due to increased drinking behaviour seems to be the most likely reason for the development of peripheral oedemas leading to an increase in the foot volume in the present runners.

A further finding was that the change in body mass was significantly and negatively related to running speed, where faster runners were losing more body mass. Similar findings reported Lebus et al. [44] for 161-km ultra-marathoners and Kao et al. [10] for 24-hour ultra-marathoners, where a greater body mass loss was associated with a better performance. Furthermore, Sharwood et al. [22] demonstrated that Ironman triathletes showing the greatest changes in body mass were among the fastest finishers. Our finding allows us to support the suggestion [10] that maintenance in body mass is not crucial to performance in ultra-endurance races. Thus, there was no evidence in our study that an increased loss in body mass impaired performance.

We were measuring the feet volume using plethysmography. The same method used Bracher et al. [32] for measuring the volumes of both the lower leg and arm in ultra-marathoners. This method using plethysmography is similar to the method from Lund-Johansen et al. [46] measuring the leg volume by using water displacement volumetry. Lund-Johansen et al. [46] concluded that the water displacement volumetry is a sensitive method for the measurement of leg volume. Therefore, we assumed that measuring changes in foot volumes using plethysmography was an accurate method as well.

**Figure 10:**
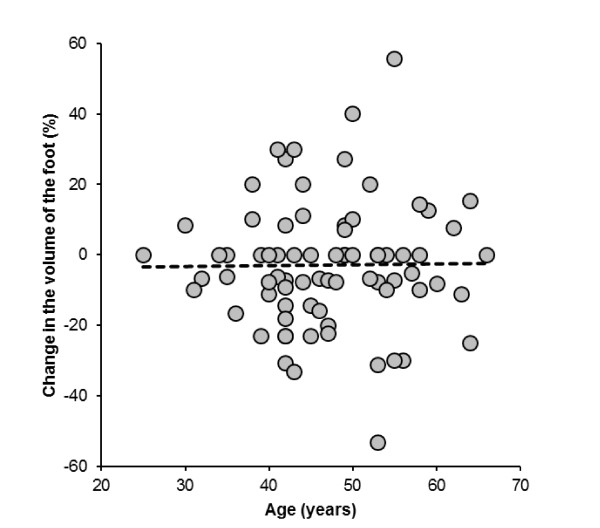
**The change in the volume of the right foot was not associated with the age of the subjects (*r *= 0.01, *p *= 0.91)**.

A limitation in our study is the fact that we did not determine total body water as it has been reported in studies investigating changes in total body water during exercise for example through the diluted isotope method [42,43]. This might provide more insight into the hydration status in ultra-marathoners, since we can only assume that total body water was increased in the slower runners leading to peripheral oedemas in these subjects. Furthermore, we did not ask our athletes about wearing compression stockings [47]. Elastic compression stockings can prevent the development of oedema in long-haul flights [48]. It would be interesting to determine in future field-studies, whether compression stockings have an influence on the development of peripheral oedemas in ultra-marathoners. The foot swelling might also be a high protein interstitial space fluid swelling and may be associated with markers of skeletal muscle damage. Leg swelling might also be due to venous insufficiency with a higher prevalence at advanced ages [49]. However, when plotting changes in foot volume versus age, we found no association between changes in foot volume and an increase in age (Figure 10).

## Conclusions

In summary, this study demonstrated that fluid intake was positively related to the volume of the foot in 100-km ultra-marathoners. An increase in the foot volume occurred in athletes with an increased fluid intake. In addition, slower running speed was associated with an increase in the foot volume and the change in foot volume was negatively correlated to the change in plasma [Na^+^]. Therefore, we concluded that fluid overload occurred in slower runners and was responsible for the development of oedemas in the foot. In addition, post-race plasma [Na^+^] decreased in those runners. Our data support the finding that fluid overload is the main risk factor for developing EAH [19-21]. For practical application, athletes performing an ultra-marathon should be aware that excessive drinking with fluid overload increases the risk for EAH [19-21] and can lead to the development of peripheral oedemas in the foot.

## Competing interests

The authors declare that they have no competing interests.

## Authors' contributions

CC wrote the manuscript, BK and PK collected the data at the race, CAR and TR assisted in data analysis, data interpretation and manuscript preparation. All authors have read and approved the final version.
